# Multidrug Resistant* Pseudomonas* Mycotic Pseudoaneurysm following Cardiac Transplant Bridged by Ventricular Assistant Device

**DOI:** 10.1155/2017/1402320

**Published:** 2017-03-13

**Authors:** C. Aye, M. Williams, R. Horvath

**Affiliations:** ^1^Infection Management Unit, The Prince Charles Hospital, Brisbane, QLD 4032, Australia; ^2^School of Medicine, University of Queensland, Brisbane, QLD, Australia; ^3^Department of Pharmacy, The Prince Charles Hospital, Brisbane, QLD 4032, Australia

## Abstract

Mycotic pseudoaneurysm of aorta following cardiac surgery is rare but is highly fatal if it is unrecognized and untreated. Here, we report a case of a 45-year-old male patient who presented with rapidly progressive multiple pseudoaneurysms of the ascending aorta infected with multidrug resistant (MDR)* Pseudomonas aeruginosa at* 5 weeks after cardiac transplantation, on a background of prior bridging therapy with left ventricular assistant device (LVAD). The patient was successfully treated with the newer cephalosporin, Ceftolozane/Tazobactam, in combination with surgery. This is the first reported case of mycotic pseudoaneurysm infected with MDR* Pseudomonas*. This case also highlights the importance of high vigilance and timely multimodality treatment in the diagnosis and management of mycotic pseudoaneurysm following cardiac transplant, especially in patients who had LVAD.

## 1. Introduction

Mycotic aneurysm is defined as any extra or intracardiac aneurysm of infectious etiology excluding syphilis. It involves aorta after open-heart surgeries such as cardiac transplant or coronary artery bypass, mostly at the anastomotic site and aortic suture lines [1]. The risk is higher with the transplant surgery probably due to the immunosuppressed state of the host and the need for extensive surgical dissection with the resultant intraoperative contamination to mediastinum [2].

## 2. Case

A 45-year-old man presented with 3-day history of fever and thoracic back pain at 5 weeks after receiving an orthotopic heart transplant for idiopathic dilated cardiomyopathy. The patient was on a left ventricular assistant device (HeartWare LVAD) as the bridging therapy for decompensated heart failure for 6 months prior to the cardiac transplant. There was no prior history of infective endocarditis or LVAD related infections such as pump, pocket, or drivelines infections. Immediate posttransplant period was unremarkable and he was discharged on standard immunosuppressive therapy with Prednisolone, Cyclosporine, and Mycophenolate.

On examination, he was alert, febrile at 38.7°C, and haemodynamically stable with blood pressure of 110/65 mmHg and pulse rate of 120 bpm. There was no spinal tenderness or other localizing sign. His sternotomy wound and the previous LVAD exit site had healed well without evidence of cellulitis. His heart sounds were dual and lungs were clear with good air entry on auscultation. There was no sign of cardiac failure. He did not have any focal neurological deficit.

Investigations showed elevated serum C-reactive protein (CRP) of 163 mg/L (ref: <5.0), elevated white cell count of 13.0 × 10^9^/L (ref: 4.0–11.0), predominantly neutrophils (12.66 × 10^9^/L) (ref: 2.0–8.0), low hemoglobin of 83 g/L (ref: 135–180), normal platelet count of 175 × 10^9^/L (ref: 140–400), urea of 14.9 mmol/L (ref: 2.1–7.1), creatinine of 119 umol/L (ref: 60–110), normal liver function test, and coagulation profile. Chest X-ray showed mild cardiomegaly but no consolidation or effusion. Urinalysis was unremarkable with negative blood and white cells.

He was initially managed with empirical intravenous (IV) Gentamicin and Piperacillin-Tazobactam. Blood cultures subsequently grew multidrug resistant* Pseudomonas aeruginosa*, which was sensitive to Gentamicin, Amikacin, and Colistin by disc testing but resistant to Meropenem, Piperacillin-Tazobactam, Ceftazidime, Cefepime, and Ciprofloxacin. Further minimum inhibitory concentration (MIC) showed MIC = 12 for Meropenem and MIC = 2 for Ciprofloxacin. The same* Pseudomonas* species was previously isolated from a routine superficial wound swab from the LVAD exit site 4 weeks prior to the current presentation. The patient was afebrile without any obvious evidence of cellulitis at that time, but one week of IV Gentamicin was administered with subsequent good healing of the exit site.

**Figure 1:**
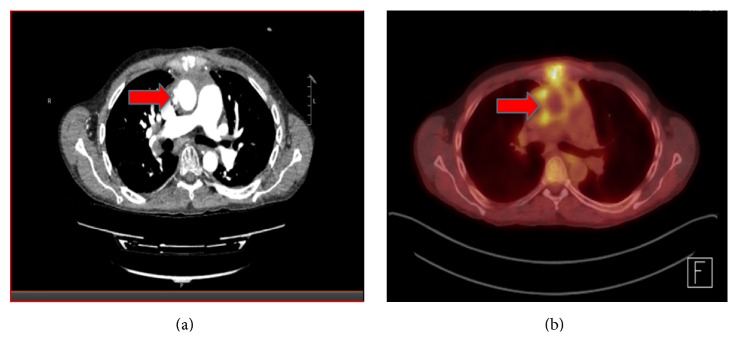
Cross-sectional views of CT angiogram image (a) and PET image (b) of the patient's chest, showing two outpouchings in the wall of ascending aorta (pointed by red arrows) which were FDG avid on the PET, indicative of mycotic pseudoaneurysms.

In order to localize the infective focus, transoesophageal echocardiogram (TOE) and CT chest/abdomen/pelvic were performed. TOE excluded infective endocarditis but revealed 2 small outpouchings, measuring 2.5 × 2.4 cm and 0.45 × 0.6 cm, in the ascending aorta, which were also seen on CT chest (Figure 1(a)). PET scan using ^18^F-fluorodeoxyglucose (FDG) tracer showed moderate FDG uptake in the aortic outpouchings and the diagnosis of mycotic pseudoaneurysm was made (Figure 1(b)).

The case was discussed with the cardiothoracic surgical team, and a trial of conservative management with IV Gentamicin and Meropenem was instituted. Gentamicin was administered once daily, targeting an area under the concentration-time curve from 0 to 24 hours of concentration (AUC_24_)/MIC ratio of 70–100 and Meropenem was administered as a continuous intravenous infusion of 6 grams over 24 hours, targeting a steady-state unbound plasma concentration above the MIC.

Assessment of clinical progress after 2 weeks of conservative treatment revealed subjective improvement and some objective response such as normalization of temperature and CRP with sterile repeat blood cultures. However, a follow-up PET/CT scan revealed interval increase in size of the aortic outpouchings, measuring 2.1 × 3.1 cm and 0.7 × 0.7 cm, and another new outpouching with significant FDG uptake associated with peripheral enhancement within left anterior mediastinum concerning focal collection. A surveillance magnetic resonance imaging (MRI) of brain also showed small areas of restricted diffusion in frontal and parietal lobes bilaterally suggestive of acute embolic infarcts although the patient did not have any focal neurological deficits.

The antibiotic therapy was changed to IV Ceftolozane/Tazobactam 1000 mg/500 mg every 8 hours and the patient proceeded to have redo-sternotomy, total excision of pseudoaneurysm, and associated ascending aorta and aortic allograft implantation. Cultures of the resected specimen grew the same MDR* Pseudomonas* and additional sensitivity test showed that it was sensitive to Ceftolozane/Tazobactam (MIC = 4). Postoperatively, antibiotic therapy was continued with IV Ceftolozane/Tazobactam for 8 weeks followed by oral Ciprofloxacin 750 mg twice daily.

The patient is now 6 months after discharge and remains well without any evidence of recurrence. The plan is to continue oral Ciprofloxacin as lifelong suppressant therapy.

## 3. Discussion

Mycotic aneurysm is defined as any extra or intracardiac aneurysm of infectious etiology excluding syphilis. It involves aorta after open-heart surgeries such as cardiac transplant or coronary artery bypass, mostly at the anastomotic site and aortic suture lines [1] The risk is higher with the transplant surgery probably due to the immunosuppressed state of the host and the need for extensive surgical dissection with the resultant intraoperative contamination to mediastinum [2]. The incidence was reported to be very low, especially in early literatures. A review in 1990 reported no known case of aortic pseudoaneurysm for over 5000 cardiac transplantation procedures performed [1] and that in 1996 reported that only 10 cases of mycotic aneurysm had occurred out of more than 30,000 cardiac transplants performed worldwide [3].

The incidence seems to have increased over the last two decades. In 2011, Tang et al. reported a series of 8 cases from their single institution and 12 other cases that were published between 1999 and 2011. Five out of the eight cases had LVAD as bridging therapy, and the authors suggested that the increasing use of bridging LVAD might lead to an increase prevalence of mycotic pseudoaneurysm following cardiac transplantation [4]. LVADs are associated with a very high risk of infection and the suture line of the outflow prosthesis of the LVAD represents a potential site for aneurysmal formation in the transplant recipients [5]. In our patient, the pseudoaneurysm may have been related to direct bacterial seeding from the previous LVAD exit site, given the same* Pseudomonas* was isolated.

Presentation with mycotic pseudoaneurysm is usually delayed after the transplant procedure, from a couple of months to years [6]. Most of the patients have preceding history of mediastinal infection [4–7]. Our patient did not have any clinical features suggestive of mediastinitis at the time of presentation, but it seemed to declare subsequently, with possible mediastinal collection on a follow-up PET-CT.

Mycotic aneurysm postcardiac transplant poses very high mortality, up to 50% [6–8] despite its low incidence. Optimal management of the condition has been debatable. However, early recognition, surgical intervention of the infected aneurysm, and adjuvant antibiotic therapy seem to be the keys to successful management of mycotic aneurysm in cardiac transplant patients. Tang et al. reported zero mortality in seven patients, and they attributed the excellent outcomes to their low index of suspicion for imaging, leading to early diagnosis, and multimodality management [4]. Other authors have also suggested careful follow-ups with blood cultures, echocardiogram, and CT/MRI, especially in patients bridged by VADs [6]. Successful outcomes required definitive surgical treatment with removal of mycotic aneurysm and vascular graft replacement and concomitant appropriate antimicrobial therapy [4, 9], as in our case. The recommended duration of antimicrobial therapy is at least 4–6 weeks of intravenous treatment [3, 7]. A lifelong course of PO regime has also been suggested as mandatory [9]. The choice of antibiotic should be based on the culture and sensitivity of the isolated organism.

Common isolated causative microorganisms include* Staphylococcus aureus*,* Candida* species, and* Pseudomonas aeruginosa* but infection with Coagulase negative* Staphylococcus*, Methicillin resistant* Staphylococcus aureus*, Vancomycin resistant* Enterococci*,* multidrug resistant Enterobacter cloacae,* and* Aspergillus* has also been reported [2–4, 7, 9]. Our patient grew multidrug resistant* Pseudomonas aeruginosa*, which has not previously been reported in the literature to our knowledge.

In choosing an appropriate antibiotic regime in our patient, microbial sensitivity (MIC), renal function, drug interactions in context of nephrotoxic immunosuppressant therapy, and practicality of therapeutic drug monitoring were taken into consideration. Meropenem was administered by continuous infusion in an attempt to optimize time above MIC (*T*_>MIC_) and to allow administration of a higher dose without peak concentrations that might result in neurotoxicity. While studies investigating clinical outcomes of administering beta-lactams by continuous infusion or extended infusion versus standard dosing have had mixed clinical outcomes [10, 11], these studies have consistently shown that administration by continuous infusion results in a higher *T*_>MIC_. Using a continuous infusion, we achieved a free fraction steady-state Meropenem concentration of 44 mg/L which was approximately 3.5 times the MIC and was tolerated without any apparent neurotoxicity. On the other hand, therapeutic drug monitoring of Gentamicin was performed by calculating an AUC as described by Begg et al. [12] using the Microsoft Excel spreadsheet validated by Wong et al. [13]. We managed to achieve an *C*_max_: MIC of >10 and an AUC_0–48 h_: MIC of 140–200 mg·h/L by dosing every 48 hours.

Despite this, medical treatment appeared to fail and the Gentamicin was substituted with IV Ceftolozane/Tazobactam. Ceftolozane is a novel bactericidal antipseudomonal cephalosporin, which exerts bactericidal activity by inhibition of bactericidal cell-wall synthesis. Tazobactam is a beta lactamase inhibitor by inhibiting certain class A and class C beta lactamase. The combination is stable against most common* Pseudomonas aeruginosa* resistance mechanisms including loss of outer membrane porin (OprD), chromosomal AmpC, and upregulation of efflux pumps (MexXY, MexAB). The use of Ceftolozane/Tazobactam has been well described in the treatment of complicated intra-abdominal infections in combination with Metronidazole and in complicated urinary tract infections including pyelonephritis [14, 15]. To our knowledge this is the first reported use of Ceftolozane/Tazobactam in an endovascular infection.

In summary, this is the first reported case of mycotic pseudoaneurysms of aorta with MDR* Pseudomonas aeruginosa* postcardiac transplant, in a patient who had bridging therapy with LVAD. The case displayed rapid progression but was successfully managed by timely surgery in conjunction with appropriate concurrent antibiotic therapy. The case highlights the importance of close follow-up, low index of suspicion for vascular imaging, early diagnosis, and multimodality intervention in cardiac transplant patients bridged by VAD.
